# Intrauterine devices migrated into the bladder: two case reports and literature review

**DOI:** 10.1186/s12905-021-01443-w

**Published:** 2021-08-16

**Authors:** Guangtao Liu, Feifei Li, Min Ao, Guimin Huang

**Affiliations:** 1 Department of Urology, People’s Hospital of Leshan, 238 BaitaStreet, Leshan, 614000 Sichuan Province People’s Republic of China; 2 Department of Oncology, People’s Hospital of Leshan, Leshan, 614000 Sichuan Province People’s Republic of China

**Keywords:** Intrauterine devices, Migration, Bladder, Lower urinary symptoms, Case report

## Abstract

**Background:**

Intrauterine devices (IUD) are widely used all over the world. One of the most serious complications is uterine perforation, and it is very rare for the IUD to penetrate the bladder after perforation. Here we report two cases of IUD migration into the bladder, and review the literature to analyze the possible causes and solutions of such complications.

**Case presentation:**

Case NO. 1 is a 37-year-old female who presented lower urinary tract symptoms for a year. Cystoscopy showed that a strip of metal penetrated into the bladder, and the surface was covered with stones. The patient underwent cystotomy and foreign body removal under general anesthesia. Case NO. 2 is a 46-year-old woman who previously inserted an IUD in 1998, but she had an unexpected pregnancy in 1999. Her doctor believed that "the IUD had spontaneously expulsed" and a new IUD was inserted after her pregnancy was terminated. Her CT scan showed an IUD on the left side of the bladder and another IUD in the uterus. Her foreign body was removed by cystotomy.

**Conclusion:**

Patients with IUD should be suggested to check the device regularly, and those who with a missed IUD have to rule out the possibility of IUD migration. For patients with IUD combined with lower urinary tract symptoms, it is necessary to be aware of whether IUD perforation affects the bladder.

## Background

As one of the main contraceptive methods, intrauterine devices (IUD) are widely used all over the world. It has a low incidence of complications and the complications are generally not severe. One of the most serious complications is uterine perforation, and it is very rare for the IUD to penetrate the bladder after perforation [1]. Here we report two cases of IUD migration to the bladder and review the literature on this issue.

### Case presentation

#### Case no. 1

**Fig. 1:**
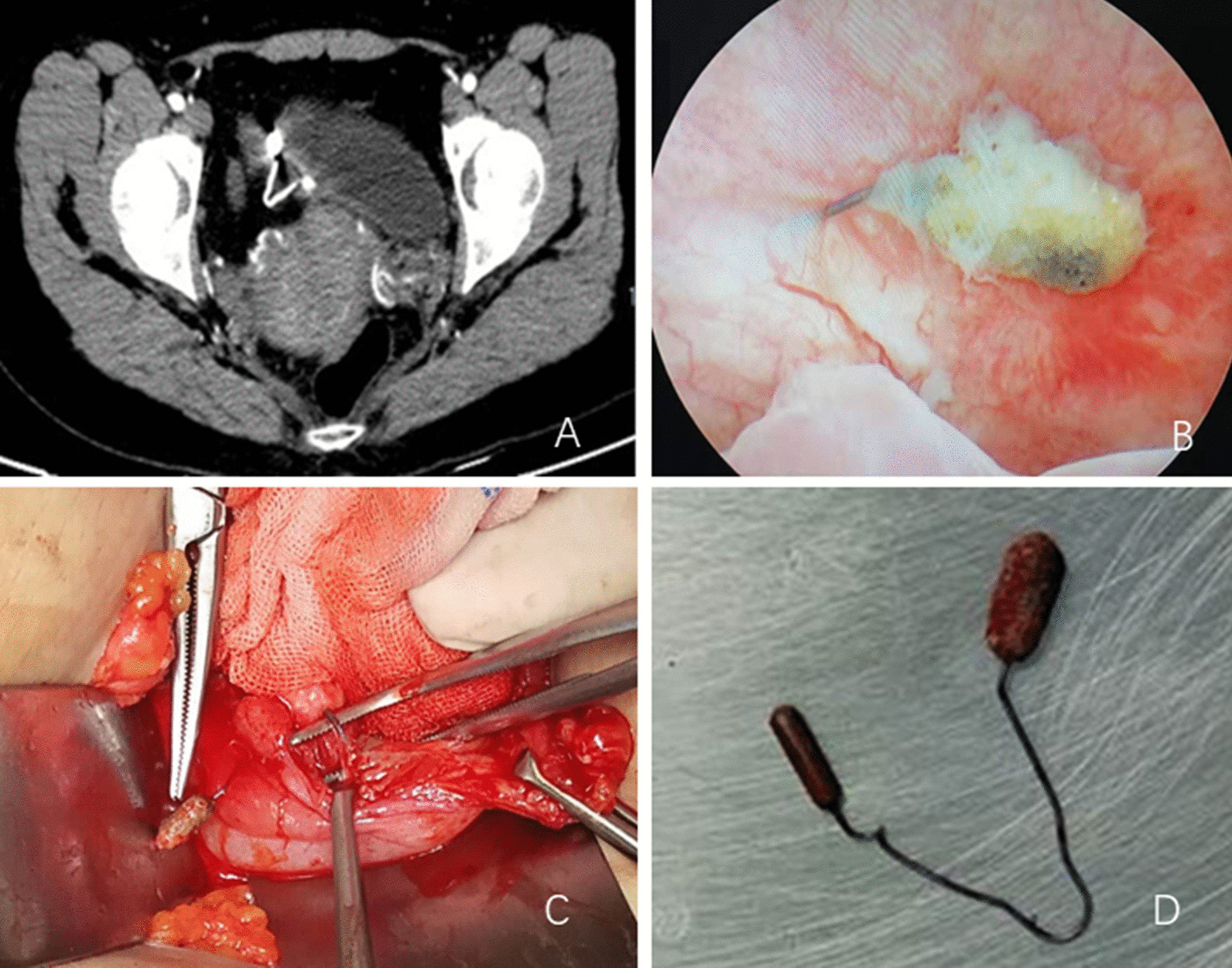
IUD of case NO.1 (**a**: CT scan indicating a triangular foreign body penetrated the bladder wall. **b**: Cystoscopy showed a strip of metal substance penetrated through the bladder wall, and the surface was covered with stones. **c**: Intraoperative photograph showed that a branch of the IUD passed through the bladder wall. **d**: The removed IUD.)

A 37-year-old female was admitted to our department presented lower urinary tract symptoms for a year, including urinary frequency, urgency, and hematuria. The patient was placed an IUD 9 years ago in the local hospital after vaginal delivery of two children. After that, she had no discomfort and no follow-up. Her physical examination was normal. Urinalysis shows WBC (−), RBC 3+. There was no bacterial growth in urine culture. Color doppler ultrasound showed that there was a hyperechoic mass in the bladder, which was probably a blood clot. The CT scan revealed a triangular foreign body at the top of the bladder, penetrating the bladder wall. Cystoscopy showed that a strip of metal substance penetrating into the right wall of the bladder, and the surface of which was covered with stones (Fig. 1). We tried to extract it under cystoscopy, but it wouldn't move. The diagnosis of this patient was bladder foreign body, bladder stone, and urinary tract infection. On August 8, 2019, the patient underwent cystotomy and foreign body removal under general anesthesia. During the operation, we found that one branch of the contraceptive device pierced into the right wall of the bladder, the other branch was located in the tissue between the uterus and the bladder.

#### Case no. 2

**Fig. 2:**
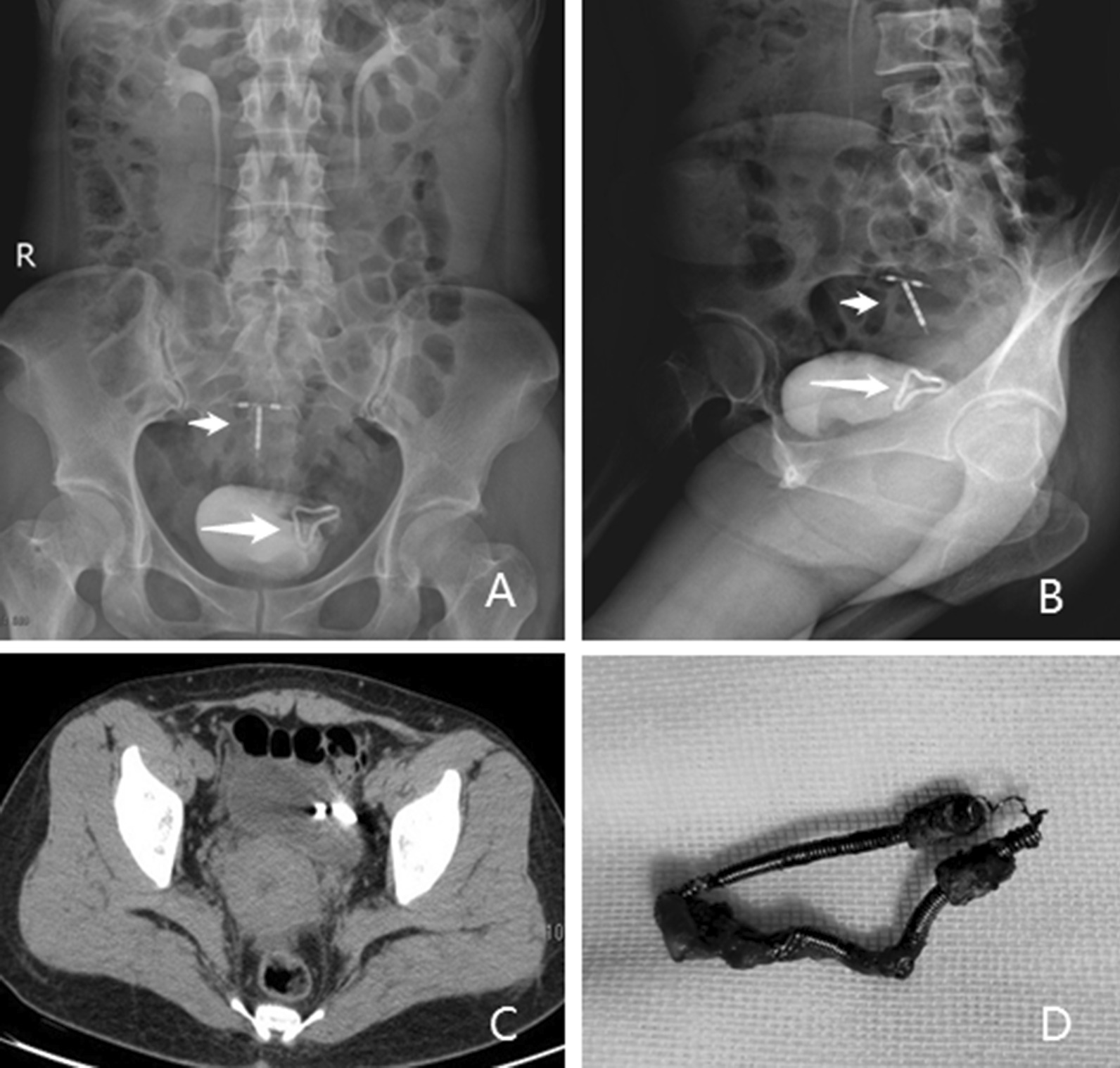
IUDs of case NO.2 (**a** and **b**: IVP demonstrating two IUDs in the pelvic cavity, one of them was in the bladder. **c**: Her CT scan showing an IUD penetrated the left side of the bladder. **d**: The removed IUD.)

A 46-year-old woman was referred to our hospital complaining of "frequent urination, urgency and pain for over 10 days". The patient had a history of three vaginal deliveries and no C-sections. She was previously placed an Intrauterine device in a local hospital in 1998, but she had an unexpected pregnancy in 1999. As no indication of a residual IUD was found in the uterus on ultrasonography, her doctor believed that "the IUD had spontaneously expulsed", and a new IUD was inserted after her abortion. Her physical examination was normal. The urinalysis showed leukocyte 1 + , and urine culture turned out to be Escherichia coli. Her CT scan showed an IUD on the left side of the bladder and another IUD in the uterus (Fig. 2). Also, her IVP films showed two IUDs in the pelvic cavity. Her cystoscopy revealed an 1.5 cm long U-shaped foreign body near the left ureteral orifice, with calculus on its surface. The posterior end of the foreign body was inserted into the bladder wall, causing local inflammation of the bladder. As the foreign body was very close to the left ureteral orifice, considering the difficulty of foreign body removal under cystoscopy or laparoscopy, we performed cystotomy to remove the foreign body for her under general anesthesia on November 12, 2019. The patient recovered well and was discharged 5 days after operation.

## Discussion and conclusions

Intrauterine devices(IUD) are widely used for their simplicity, low cost, safety, effectiveness, and reversibility. Common complications of IUD include dysmenorrhea, hypermenorrhea, bleeding, pain, and pelvic infection. The incidence of IUD migration is very low, ranging from 0.1 to 0.9% as reported in the literature [2]. During the puerperium and lactation period, the uterine wall is thin and soft, and the possibility of IUD migration is the greatest. According to the literature, its risk factors include previous history of caesarean section, but neither of our two patients had a history of caesarean section, so it may be affected by other factors, such as the experience and proficiency of the practitioner. At present, there is no reliable data to confirm whether the type and material of IUD can affect the IUD migration, and further research is needed. Perforation is more likely to occur in the early stage or immediately after IUD placement. In the presence of difficulty insertion, pain, or bleeding, the doctors should be alert to the possibility of acute perforation [3–5]. Our case NO.2 became pregnant shortly after the placement of the IUD, and presumably had a displacement early after that. Bjornerem reported a case in which the patient apparently had difficulty insertion and associated pain. One week after the IUD placement, symptoms such as lower abdominal pain and frequent urination appeared. Three weeks later, cystoscopy revealed that the IUD was completely transferred to the bladder, with intact bladder Mucosa and no signs of perforation [6]. This demonstrates that IUDs can migrate to the bladder in a short period of time.

However, IUDs usually do not cause any discomfort when they pass through the uterus chronically, and in most cases they do not affect other organs. It only causes symptoms when it enters the abdominal cavity, punctures the intestine or other organs. Only 2% of the displaced IUDs may affect the bladder [6]. After passing through the bladder wall, it often leads to bladder irritation symptoms, and stones will form over time. Its common symptoms include frequent urination, urgency, dysuria, hematuria, and lower abdominal pain, etc. These symptoms were typical in both of our cases.

Schwartzwald reported a case of uterovesical fistula and menstrual hematuria due to the perforation of IUD. The patient did not recover until the uterus was removed and fistula was repaired [7]. However, some patients had no obvious discomfort, and it was not found that the IUD had migrated until pregnancy. Some cases also fail to find that the IUD had displaced during the prenatal care, so it is assumed that the IUD has expulsed spontaneously, and some even insert a new IUD soon, just like our case NO.2. It is possible that during the ultrasound examination of this patient, the physician focused only on intrauterine conditions and did not fully explore the pelvic cavity, resulting in the missed diagnosis of ectopic IUD. Although 20% of patients with spontaneous discharge are unaware expulsion, they must be confirmed with a negative abdominal plain film or see the discharged IUD in person, so as to avoid serious consequences due to missed diagnosis [6].

How to remove the IUD in the bladder requires a reasonable choice based on its position, shape, patient conditions, and hospital equipment [8, 9]. If the IUD is partially perforated and the string is still in the vagina, try to remove it through the vagina. Kiilholma reported a case in which her IUD had partially penetrated the bladder, but strings remained in the cervix. The IUD was successfully removed through vagina by string extraction [10]. If the IUD is completely or mostly in the bladder, it can be removed by cystoscopy. Most of the cases reported in the literature are solved in this way. If part of the IUD is intraperitoneal, it may be removed by laparoscopy, or laparoscopy combined with cystoscopy. However, if these methods are difficult to remove, open surgery is required. For the two cases reported in this article, a portion of the IUDs were embedded in bladder wall and calculus was formed. It was difficult to remove IUDs by minimally invasive surgery, so open surgery was performed.

In conclusion, patients with IUD should be suggested to check the device regularly, and those who with a missed IUD have to perform the abdominal pelvic X-ray to rule out the possibility of IUD migration. For patients with IUD combined with lower urinary tract symptoms such as frequent urination, urgency, and hematuria, it is necessary to be aware of whether IUD perforation affects the bladder. As such patients are relatively rare, it is very important for urologists and obstetricians to have this awareness.

## Data Availability

All data related to this case report are available from the corresponding author on reasonable request.

## Abbreviations

IUD: Intrauterine devices
CT: Computed Tomography
WBC: White blood cells
RBC: Red blood cells
IVP: Intravenous pyelography
